# Risk of invasive breast cancer in relatives of patients with breast carcinoma in situ: a prospective cohort study

**DOI:** 10.1186/s12916-020-01772-x

**Published:** 2020-11-05

**Authors:** Trasias Mukama, Mahdi Fallah, Hermann Brenner, Xing Xu, Kristina Sundquist, Jan Sundquist, Elham Kharazmi

**Affiliations:** 1 Division of Preventive Oncology, Risk Adapted Prevention (RAD) Group, German Cancer Research Center (DKFZ) and National Center for Tumor Diseases (NCT), Im Neuenheimer Feld 581, 69120 Heidelberg, Germany; 2 Medical Faculty Heidelberg, University of Heidelberg, Heidelberg, Germany; 3 Department of Disease Control and Environmental Health, School of Public Health, College of Health Sciences, Makerere University, Kampala, Uganda; 4 Center for Primary Health Care Research, Lund University, Malmö, Sweden; 5 Division of Clinical Epidemiology and Aging Research, German Cancer Research Center (DKFZ), Heidelberg, Germany; 6 German Cancer Consortium (DKTK), German Cancer Research Center (DKFZ), Heidelberg, Germany; 7 Department of Family Medicine and Community Health, Department of Population Health Science and Policy, Icahn School of Medicine at Mount Sinai, New York, USA; 8 Center for Community-based Healthcare Research and Education (CoHRE), Department of Functional Pathology, School of Medicine, Shimane University, Izumo, Japan; 9 Statistical Genetics Group, Institute of Medical Biometry and Informatics, University Hospital Heidelberg, Heidelberg, Germany

**Keywords:** Breast cancer, Breast carcinoma in situ, Mammography screening, Familial breast cancer

## Abstract

**Background:**

Wide implementation of mammography screening has resulted in increased numbers of women diagnosed with breast carcinoma in situ. We aimed to determine the risk of invasive breast cancer in relatives of patients with breast carcinoma in situ in comparison to the risk in relatives of patients with invasive breast cancer.

**Methods:**

We analyzed the occurrence of cancer in a nationwide cohort including all 5,099,172 Swedish women born after 1931 with at least one known first-degree relative. This was a record linkage study of Swedish family cancer datasets, including cancer registry data collected from January 1, 1958, to December 31, 2015. We calculated standardized incidence ratios (SIRs) and 10-year cumulative risk of breast cancer diagnosis for women with a family history of in situ and invasive breast cancer.

**Results:**

Having one first-degree relative with breast carcinoma in situ was associated with 50% increased risk of invasive breast cancer (SIR = 1.5, 95% CI 1.4**–**1.7) when compared to those who had no family history of invasive breast cancer or breast carcinoma in situ in either first- or second-degree relatives. Similarly, having one first-degree relative with invasive breast cancer was associated with 70% (1.7, 1.7**–**1.8) increased risk. The 10-year cumulative risk for women at age 50 with a relative with breast carcinoma in situ was 3.5% (2.9**–**3.9%) and was not significantly different from 3.7% (3.6**–**3.8%) risk for 50-year-old women with a relative with invasive breast cancer (95% confidence intervals overlapped).

**Conclusions:**

The risk of invasive breast cancer for women with a family history of breast carcinoma in situ was comparable to that for women with a family history of invasive breast cancer. Therefore, family history of breast carcinoma in situ should not be overlooked in recommendations for breast cancer prevention for women with a family history of breast cancer.

## Background

Breast cancer is a major cause of cancer morbidity and mortality worldwide, accounting for a quarter of all new cancer cases [1]. Many high-income countries initiated nationwide breast cancer screening programs following numerous studies that indicated association of mammography screening with reduction in breast cancer mortality [2, 3]. Effective breast cancer screening enables detection and treatment of early-stage cancer and thus provides the possibility to prevent an advanced form of this disease. However, implementation of population-wide screening programs has resulted in an increased number of women diagnosed with breast carcinoma in situ [4, 5] and thus an increasing number of women reporting a family history of breast cancer and breast carcinoma in situ [6].

Breast carcinoma in situ, also known as stage 0 breast carcinoma or intraductal carcinoma, is defined by the presence and proliferation of malignant cells that remain confined within the breast duct above the basement membrane without spreading through the walls of the ducts into the nearby breast tissue [7]. The majority of predisposing genetic changes linked to invasive breast cancer are also linked to in situ disease, supporting the view that breast carcinoma in situ and invasive carcinoma may represent different phases of the same disease [8].

Having a family history of invasive breast cancer is known to be associated with an increased risk of the disease [9, 10]. Indeed, breast cancer screening guidelines, such as those issued by the American College of Radiology, recommend earlier screening initiation for women with family history of breast cancer [11]. These and other guidelines however lack recommendations for family members of patients with breast carcinoma in situ, and it remains unclear whether the recommendations for women with family history of invasive breast cancer are applicable to women with family history of breast carcinoma in situ or not. In this study, we aimed to determine the risk of invasive breast cancer for women with a family history of breast carcinoma in situ, using nationwide Swedish family cancer data sources. These datasets are the world’s largest of their kind and consist of long-term register-based genealogy and pathologically verified cancer registry data, which provide a unique opportunity to investigate this question.

## Methods

### Study design

This study used an ambidirectional design including both retrospective (family history) and prospective (biannual update of cancer status) components of the study, in which a cohort of Swedish women born after 1931 was followed up from 1958 onwards for invasive breast cancer diagnosis through record linkage. The datasets and thus the exposure and outcome of interest are updated biannually. For this study, we used the latest version available with follow-up by the end of 2015.

### Setting

This study was based on record linkage of several nationwide Swedish datasets: The Multi-Generation Register, which is a genealogy dataset, in which families comprising of offspring (born after 1931) are linked to their parents. The Multi-Generation Register was linked with the Swedish Cancer Registry (started in 1958) using unique personal identification numbers. The personal identifiers have been pseudonymized and replaced with technical serial numbers to prevent the identification of individuals in the datasets. The linkage and structure of the linked datasets have been previously described elsewhere [12, 13]. The registration of newly diagnosed cancers is based on mandatory reporting by all physicians in public and private facilities, and the data are supplemented with information on cause and date of death through linkage with the Swedish Cause of Death Register. The cancer registry is estimated to be 97% complete, including registration of breast carcinoma in situ [14–16]. Within the register, cancers were identified through a four-digit code according to the 7th revision of the International Classification of Disease (ICD-7) although codes based on later revisions are also available.

The latest version of family cancer datasets (with information by the end of 2015), which was updated in 2017, was used in this study and includes records of more than 12.8 million individuals with genealogical data, about 1.7 million records of primary invasive cancers, and around 0.5 million records of carcinomas in situ. The behavior of breast cancer tumors (in situ or invasive) had been defined by the cancer registry based on the 3rd digit of the Pathological Anatomic Diagnosis (PAD) code (value “4” for in situ and “6” for invasive) from 1958 to 1992 and 5th digit of morphology code in the second/third version of International Classification of Diseases coding system for Oncology (ICD-O-2/3; value “/2” for in situ and “/3” for invasive) from 1993 onward. Both ductal and lobular breast carcinoma in situ were included. Further information on the grade of carcinoma in situ was not available. We also used longitudinal demographic and socio-economic data from national censuses. Data from January 1, 1958, to December 31, 2015, were collected. Data were analyzed from October 1, 2018, to January 31, 2020.

### Participants

All women resident in Sweden and born after 1931 (the offspring generation) were eligible for the study. We restricted the current analysis to the offspring generation to minimize possible birth cohort effects because we found lower incidence rates of breast cancer in the parental generation. Through the linked datasets, it was possible to establish the numbers of both first-degree and second-degree relatives affected with invasive breast cancer or breast carcinoma in situ diagnosis, including their ages at diagnosis. Out of 6,332,770 women with genealogical data, 5,099,172 women who were born after 1931 were included in our analysis. Participants were followed up from birth, year of immigration, or the starting year of cancer registry (1958), whichever came latest, and follow-up ended at year of invasive breast cancer diagnosis, year of death, year of emigration, or end of the study (2015), whichever came earliest.

### Statistical analysis

The standardized incidence ratio (SIR) was used to compare cancer risk according to family history status of invasive and breast carcinoma in situ. The SIRs were calculated as ratios of the observed (O) to the expected (E) number of cases. It has been reported that using the disease incidence in the entire population as an approximate measure of the disease incidence in the unexposed population, which is a common practice in SIR calculation, results in a bias towards the null, the magnitude of which depends on the prevalence of the exposure and the magnitude of the true relative risk [17]. The opportunity to clearly define exposed (women with family history of invasive breast cancer or family history of breast carcinoma in situ) and unexposed groups (women without family history of either invasive breast cancer or breast carcinoma in situ) allowed elimination of any such bias. The expected rates were calculated based on strata-specific standard incidence rates in the reference group (e.g., women with no family history of invasive breast cancer or breast carcinoma in situ in first-degree and second-degree relatives). The stratification was based on age (18 groups of 5-year bands), sex, period (11 groups of 5-year bands), region, and socio-economic status (six groups: farmer, manual worker, low- to middle-income office worker, high-income office worker/professional, company owner [except farmer], and other/unspecified). The expected number of cases was calculated by multiplying strata-specific incidence rate in the reference population by the corresponding number of person-years of follow-up accumulated by individuals with a family history of interest (e.g., having one second-degree relative with breast carcinoma in situ). The 95% confidence intervals (CIs) were calculated assuming a Poisson distribution. The analyses were also stratified by age at diagnosis of invasive breast cancer in index women (age < 50 years, i.e., mostly premenopausal, or ≥ 50 years, i.e., mostly postmenopausal) and by family history of breast cancer to identify possible effects on early-onset invasive breast cancer. Family history of breast cancer was assessed as a dynamic variable and changed every time a new in situ or invasive breast cancer diagnosis occurred in a woman’s first- and second-degree relatives. Using dynamic family history should yield more appropriate familial risk estimates for informing cancer prevention interventions [18]. Family history of breast cancer from male relatives was also included.

The 10-year cumulative risk of invasive breast cancer in women with family history of breast carcinoma in situ or invasive breast cancer was calculated as follows: the age-specific incidence rate at a certain age A was calculated as the number of cases at age A divided by available person-years of follow-up at that age. The sum of 10 consecutive 1-year age-specific incidence rates from age A provided the 10-year cumulative incidence rate at that age. The 10-year cumulative rate was then converted into a 10-year cumulative risk using the following formula: 10-year cumulative risk = 100 × (1 − *e*^−(10-year cumulative rate)^). Similarly, the lifetime cumulative rate was calculated as the sum of age-specific incidence rates from birth to age 79 years. The lifetime risk was derived from the lifetime cumulative rate using the formula: lifetime cumulative risk = 100 × (1 − *e*^−(lifetime cumulative rate)^). All analyses were conducted in SAS software version 9.4 (SAS Institute Inc., Cary, NC, USA).

## Results

### Cohort description

During the study period, 5,099,172 women were followed up. The majority of the women (87.59%; *n* = 4,466,577) did not have a family history of in situ or invasive breast cancer in first- and second-degree relatives by the end of the study. Among 40,352 women with family history of only breast carcinoma in situ in first-degree and second-degree relatives, 584 developed invasive breast cancer during the follow-up.

### Risk by family history constellation

In women of all ages, having one first-degree relative with breast carcinoma in situ was associated with about 50% increased risk of breast cancer (SIR = 1.5, 95% CI 1.4–1.7) compared to having no family history of in situ or invasive breast cancer in first-degree and second-degree relatives (Table 1). In comparison, having one first-degree relative with invasive breast cancer was associated with 1.7-fold (1.7, 1.7–1.8) increased risk of invasive breast cancer and the risk was 2-fold (2.0, 1.9–2.1) increased in women under age 50 years. The risk of breast cancer for women with a first-degree relative with in situ tumor was also increased for younger women below age 50 years (1.6, 1.3–1.9). Compared to women with no family history of breast cancer in first-degree and second-degree relatives, women who had only a second-degree relative with breast carcinoma in situ were at a 20% increased risk (1.2, 1.0–1.4), whereas the risk was 30% (1.3, 1.3–1.4) increased when a second-degree relative had an invasive breast cancer. A similar risk was observed for the following two groups of women: those who had either two second-degree relatives with invasive disease (1.6, 1.4–1.8) or one first-degree relative with in situ disease (1.5, 1.4–1.7). When breast carcinoma in situ was set as the outcome of interest, similar SIRs between women with a history of breast carcinoma in situ in one first-degree relative (1.7, 1.4–2.1) and women with history of invasive breast cancer in one first-degree relative (1.7, 1.7–1.8) were found (further results by age at diagnosis are presented in Additional file 1: Table S1 and eResults section).

**Table 1:** Relative risk of breast cancer in women with family history of in situ and invasive breast cancer in first-degree and second-degree relatives

Family history of breast tumor	Age at invasive breast cancer diagnosis in index woman (years)								
	All ages			< 50			≥ 50		
	Obs	SIR	95% CI	Obs	SIR	95% CI	Obs	SIR	95% CI
**No family history of in situ or invasive**	102,177	Reference		31,693	Reference		70,484	Reference	
**1 FDR in situ**	575	**1.5**	1.4–1.7	126	**1.6**	1.3–1.9	449	**1.5**	1.4–1.7
**1 FDR invasive**	11,990	**1.7**	1.7–1.8	2874	**2.0**	1.9–2.1	9116	**1.7**	1.6–1.7
**1 SDR in situ**	132	**1.2**^§^	1.0–1.4	67	1.1	0.9–1.4	65	**1.3**	1.0–1.7
**1 SDR invasive**	2309	**1.3**^§^	1.3–1.4	1478	**1.3**	1.3–1.4	831	**1.3**	1.2–1.4
**1 SDR invasive + 1 SDR in situ**	44	**2.4**	1.7–3.2	32	**2.4**	1.6–3.4	12	**2.3**	1.2–4.1
**2 SDRs invasive**	223	**1.6**	1.4–1.8	180	**1.7**	1.5–2.0	43	1.2	0.9–1.6
**1 FDR in situ + 1 SDR invasive**	33	**2.2**	1.5–3.1	24	**2.8**	1.8–4.2	9	1.3	0.6–2.6
**1 FDR invasive + 1 SDR invasive**	453	**2.3**	2.1–2.5	248	**2.6**	2.3–2.9	205	**2.0**	1.8–2.3
**1 FDR invasive + 1 FDR in situ**	89	**2.6**	2.1–3.2	9	**3.5**	1.6–6.6	80	**2.5**	2.0–3.1
**2 FDRs invasive**	689	**2.7**	2.5–2.9	86	**4.6**	3.7–5.7	603	**2.6**	2.4–2.8

All SIRs were adjusted for age, socio-economic status, period, and region. Bold values are statistically significant

*Obs* observed number of breast cancer cases, *SIR* standardized incidence ratio, *FDR* first-degree relative, *SDR* second-degree relative, *CI* confidence interval

^§^Example: Having one second-degree relative with breast carcinoma in situ was associated with a 20% increased risk of invasive breast cancer, which was comparable to 30% increase in risk associated with having a second-degree relative with invasive breast cancer

### Risk by type and age at diagnosis of affected relative

Overall, the relative risk associated with having a sister with breast carcinoma in situ at any age (1.5, 1.3–1.7) was similar to the risk associated with having an affected mother with breast carcinoma in situ (1.5, 1.3–1.7) and was comparable to when either relative had invasive breast cancer (Table 2). However, the relative risk was higher for young women (< 50 years) with an affected sister as compared to young women (< 50 years) with an affected mother. The difference in relative risk of breast cancer in young women with an affected sister versus an affected mother did not differ by age at diagnosis of the affected relative. The risk of breast cancer for women aged 50 years and older with a sister affected with breast carcinoma in situ was 50% increased (1.5, 1.3–1.7), which was similar to the risk in those with an affected mother (1.5, 1.3–1.8).

**Table 2:** Relative risk of breast cancer by age at diagnosis in sisters and mothers

Age at diagnosis in index woman (years)	Age at diagnosis in relative (years)	Affected first-degree relative											
		Sister						Mother					
		In situ			Invasive			In situ			Invasive		
		Obs	SIR	95% CI	Obs	95% CI		Obs	SIR	95% CI	Obs	SIR	95% CI
**All ages**	**All ages**	251	**1.5**	1.4–1.7	2962	**1.8**	1.7–1.9	266	**1.5**	1.3–1.7	8456	**1.8**	1.7–1.8
	**< 50**	92	**1.7**	1.4–2.1	1135	**1.9**	1.8–2.0	22	1.3	0.8–2.0	1175	**2.2**	2.1–2.3
	**≥ 50**	159	**1.5**	1.2–1.7	1827	**1.7**	1.7–1.8	244	**1.5**	1.3–1.7	7281	**1.7**	1.7–1.8
**< 50**	**All ages**	32	**2.2**	1.5–3.1	356	**2.4**	2.2–2.7	93	**1.4**	1.1–1.7	2490	**2.0**	2.0–2.1
	**< 50**	21	**2.1**	1.3–3.2	265	**2.4**	2.1–2.7	9	1.0	0.5–1.9	625	**2.8**	2.6–3.0
	**≥ 50**	11	**2.6**	1.3–4.6	91	**2.5**	2.0–3.1	84	**1.5**	1.2–1.8	1865	**1.9**	1.8–2.0
**≥ 50**	**All ages**	219	**1.5**	1.3–1.7	2606	**1.8**	1.7–1.8	173	**1.5**	1.3–1.8	5966	**1.7**	1.6–1.7
	**< 50**	71	**1.6**^**§**^	1.3–2.0	870	**1.8**^**§**^	1.7–2.0	13	1.7^**§**^	0.9–2.9	550	**1.8**^**§**^	1.7–2.0
	**≥ 50**	148	**1.4**	1.2–1.7	1736	**1.7**	1.6–1.8	160	**1.5**	1.3–1.8	5416	**1.7**	1.6–1.7

Notes: The reference group included women with no family history of both in situ and invasive breast cancer in first- and second-degree relatives. All SIRs were adjusted for age, sex, socio-economic status, period, and region. Bold SIRs are statistically significant

*Obs* observed number of breast cancer cases, *SIR* standardized incidence ratio, *CI* confidence interval

^§^Example: For women older than 50 years, having one sister or a mother diagnosed with breast carcinoma in situ after age 50 years was associated with 60% and 70%, respectively, both of which were comparable to risk associated with either relative diagnosed before age 50 years with invasive breast cancer

### Sensitivity analysis

We did a stratified analysis by period, 1958–1994 and 1995–2015, corresponding to the period before or during scale up of mammography screening and the period after attaining nationwide coverage in Sweden. In the 1958–1994 period, the relative risk of breast cancer in relatives of patients with breast carcinoma in situ was 2-fold (2.0, 1.5–2.8) and that of relatives of patients with invasive breast cancer was 80% increased (1.9, 1.8–2.0) compared to women without family history (Table 3). During the era of nationwide mammography screening, the risk increase in relatives of patients with breast carcinoma in situ was attenuated to 1.5-fold (1.5, 1.4–1.6). Overall, the relative risk of invasive breast cancer in relatives of women with invasive breast cancer and women with breast carcinoma in situ was rather comparable (with overlapping 95% confidence intervals) in both periods before and after nationwide implementation of mammography screening.

**Table 3:** Relative risk of breast cancer for family members of patients with invasive and in situ breast tumor by period before and after nationwide coverage with mammography screening

		Age at breast cancer diagnosis in index woman (years)								
Period	Family history	All ages			< 50			≥ 50		
		***N***	SIR	95% CI	***N***	SIR	95% CI	***N***	SIR	95% CI
**1958–2015**^**a**^	**No family history**	102,177	Reference		31,693	Reference		70,484	Reference	
	**1 FDR with in situ**	575	**1.5**	1.4–1.7	126	**1.6**	1.3–1.9	449	**1.5**	1.4–1.7
	**1 FDR with invasive**	11,990	**1.7**	1.7–1.8	2874	**2.0**	1.9–2.1	9116	**1.7**	1.6–1.7
**1958–1994**^**b**^	**No family history**	21,139	Reference		14,113	Reference		7026	Reference	
	**1 FDR with in situ**	42	**2.0**	1.5–2.8	24	**1.9**	1.2–2.8	18	**2.3**	1.3–3.6
	**1 FDR with invasive**	1381	**1.9**	1.8–2.0	884	**1.9**	1.8–2.0	497	**1.8**	1.6–1.9
**1995–2015**^**c**^	**No family history**	81,038	Reference		17,580	Reference		63,458	Reference	
	**1 FDR with in situ**	533	**1.5**	1.4–1.6	102	**1.5**	1.2–1.8	431	**1.5**	1.4–1.7
	**1 FDR with invasive**	10,609	**1.7**	1.7–1.7	1990	**2.0**	2.0–2.1	8619	**1.6**	1.6–1.7

All SIRs were adjusted for age, socio-economic status, period, and region

*FDR* first-degree relative, *SIR* standardized incidence ratio, *CI* confidence interval

^a^Entire period screening

^b^Before and during scale up of mammography

^c^After nationwide coverage of mammography screening

### Absolute risk measures

**Fig. 1:**
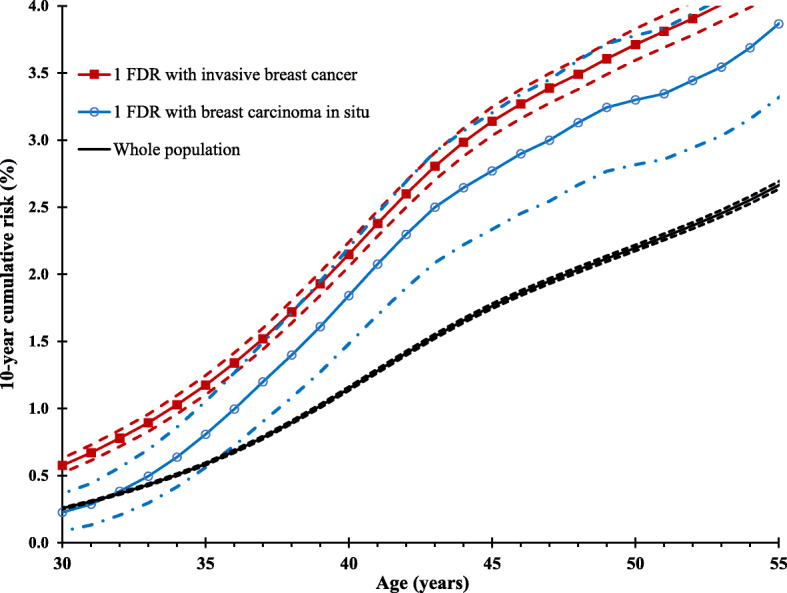
Ten-year cumulative risk curves for women with family history of breast carcinoma in situ and invasive breast cancer in one first-degree relative (FDR first-degree relative; dashed lines represent lower and upper bounds of 95% confidence interval)

At age 50 years, the 10-year cumulative risk (risk of developing breast cancer in the next 10 years) for women in the general population was 2.2% (95% CI 2.2–2.2%; Table 4). The 10-year cumulative risk for a woman at age 50 with a first-degree relative with breast carcinoma in situ was 3.4% (2.9–3.9%) and was not significantly different from 3.7% (3.6–3.8%) for women with a first-degree relative with invasive breast cancer. However, significant differences in the 10-year cumulative risk for women with family history of in situ and invasive breast cancer were observed at younger ages. For example, at age 30 years, the 10-year cumulative risk of breast cancer for a woman with family history of breast carcinoma in situ in a first-degree relative was 0.2% (0.1–0.3%), but it was 0.6% (0.5–0.6%) for women with family history of invasive breast cancer in a first-degree relative. We also showed how 10-year cumulative risk for women with family history of invasive and in situ breast tumors varies with age (Fig. 1). Although the 10-year cumulative risk for women with family history of invasive breast cancer was consistently higher than that for women with family history of in situ cancer, the 95% confidence limits overlapped from about age 35 onwards.

**Table 4:** Age-specific 10-year cumulative risk of breast cancer (%) by age at diagnosis of a first-degree relative with in situ or invasive breast cancer

Age of index woman (years)	Whole population		No family history*		Age of relative at breast cancer diagnosis (years)											
					All ages				< 50				≥ 50			
					In situ		Invasive		In situ		Invasive		In situ		Invasive	
	CR	95% CI	CR	95% CI	CR	95% CI	CR	95% CI	CR	95% CI	CR	95% CI	CR	95% CI	CR	95% CI
**30**	0.2	0.2–0.3	0.2	0.2–0.2	0.2	0.1–0.3	0.6	0.5–0.6	0.3	0.0–0.6	0.8	0.7–0.9	0.2	0.0–0.3	0.5	0.4–0.6
**35**	0.6	0.6–0.6	0.5	0.5–0.6	0.8	0.5–1.0	1.1	1.1–1.2	1.0	0.4–1.5	1.5	1.4–1.7	0.7	0.4–0.9	1.1	1.0–1.2
**40**	1.1	1.1–1.2	1.1	1.1–1.1	1.8	1.4–2.1	2.1	2.1–2.2	1.7	1.0–2.4	2.8	2.6–3.0	1.8	1.4–2.2	2.1	2.0–2.2
**45**	1.8	1.8–1.8	1.7	1.6–1.7	2.8	2.3–3.2	3.2	3.1–3.3	2.0	1.2–2.7	3.8	3.5–4.0	3.1	2.5–3.6	3.2	3.1–3.3
**50**	2.2	2.2–2.2	2.1^§^	2.0–2.1	3.4^§^	2.9–3.9	3.7	3.6–3.8	2.8	1.9–3.7	4.1	3.9–4.4	3.6	3.0–4.2	3.8	3.6–3.9
**55**	2.6	2.6–2.7	2.5	2.5–2.5	3.9	3.4–4.5	4.2	4.1–4.3	4.9	3.6–6.2	4.6	4.3–4.9	3.6	3.0–4.2	4.3	4.2–4.5
**60**	3.3	3.2–3.3	3.1	3.0–3.1	4.6	3.9–5.2	5.0	4.8–5.1	5.9	4.4–7.4	5.3	4.9–5.7	4.2	3.4–4.9	5.1	4.9–5.3
**65**	3.6	3.6–3.7	3.4	3.4–3.5	5.2	4.4–6.0	5.6	5.4–5.7	5.8	4.2–7.5	6.1	5.6–6.6	4.9	4.0–5.9	5.6	5.4–5.8

*CR* 10-year cumulative risk (%)

*No family history includes women who did not have any first-degree or second-degree relative with in situ or invasive breast cancer

^§^Example: The 10-year cumulative risk of a woman at age 50 years with a relative diagnosed with breast carcinoma in situ was 3.4%, which is higher than the risk for her peer without such a family history (2.1%)

## Discussion

We found that the risk of breast cancer was increased in women with first-degree and/or second-degree relatives with breast carcinoma in situ and it was comparable to the risk of relatives of patients with invasive breast cancer. This was observed both in the era before and after nationwide implementation of mammography screening. The 10-year cumulative risks (risk of developing breast cancer in the subsequent 10 years) were also comparable between women with family history of in situ and those with invasive breast cancer, except at young ages (less than 35 years).

Our study benefited from several register-based nationwide Swedish family cancer datasets linked together, the world’s largest of their kind, with structures that allow for accurate assessment of family history constellation of breast cancer and breast carcinoma in situ. Specifically, the use of medically verified cancer registry data enabled accurate differentiation between family history of in situ and invasive breast cancer, which is likely to be misreported in studies using patient-reported data [19]. The large sample size accorded by the Swedish family cancer datasets allowed for precise estimates for familial risk of breast cancer. In this study, family history of breast cancer was assessed as a dynamic variable—changing women’s family history every time a new family member was diagnosed, or an existing affected family member upstaged from in situ to invasive disease. Limited data are available on precise familial risks based on large-scale cohort studies from outside Sweden. However, based on similarities found for invasive cancers [20], we believe that our estimates for familial risks of breast carcinoma in situ are likely to be generalizable to populations with approximately similar cancer incidence and pattern. We adjusted for geography and socio-economic factors, which may to some extent, reconcile differences related to lifestyle.

Although risk estimates derived from Swedish cancer datasets are generally precise, there is a possibility of bias, which may arise if the diagnosis of cancer in a woman’s relatives occurred before the start of the population-based registers resulting in misclassification of family history. However, a previous study showed that for breast cancer, risk estimates from the Swedish multi-generational cohort do not generally seem to be biased by left truncation [21]. Bias may also result from differential participation in mammography screening programs by relatives of invasive and breast carcinoma patients, more so for women whose relatives were diagnosed at a younger age. A study among Swedish women however found no differential participation in future intent to participate in mammography screening [22]. Thus, such a surveillance bias is likely minimal and would not significantly alter the conclusions of the study. We did not have data on BRCA1/2 mutations to check if there is any difference in the distribution of these deleterious mutations (and possibly difference in risk of invasive breast cancer) between women with family history of invasive breast cancer and those with family history of breast carcinoma in situ. However, the effect of such differential distribution of BRCA mutations on our results is likely to be minimal as only about 10% of Swedish women with family history of invasive breast cancer have been reported to have a BRCA1/2 mutation [23].

Many studies have provided familial risks of breast cancer, mainly either as invasive or mixed with in situ family history mainly in first-degree relatives [6, 9, 10, 24, 25]. We investigated familial risks in first-degree and second-degree relatives exclusively based on family history of breast carcinoma in situ. An earlier analysis of trends in the risk of breast cancer associated with family history found no dilution of the effect of family history despite the increase in number of women reporting family history and increased incidence of breast carcinoma in situ [6].

Similar genetic variants have been shown to predispose to increased risk of both in situ and invasive breast cancer [26]. A study among Japanese women found a similar prevalence of high penetrance BRCA1/2 variants in women with invasive and breast carcinoma in situ [27]. Some studies have also demonstrated that both in situ and invasive breast cancer have shared low-penetrance genetic polymorphisms and susceptibility loci [8, 28]. Our study suggests comparability of inherited genetic predisposition and/or shared environmental risk factors for breast carcinoma in situ and invasive breast cancer at a population level.

Implementation of mammography screening has resulted in increased incidence of breast cancer, particularly of in situ tumors. Because this increase in incidence has been without a commensurate decrease in incidence of late-stage tumors, it has been deduced that most increase in incidence of in situ tumors was due to detection of indolent tumors and considered as overdiagnosed [29]. However, implementation of a breast cancer screening program has been associated with about 40% reductions in breast cancer mortality in women who participate in screening programs [30, 31]. Sweden introduced mammography screening guidelines for women 40–74 years in 1986 and attained national coverage by 1997 with high participation rates of above 70% [32]. In a stratified analysis focusing on the period after Sweden attained nationwide mammography roll-out, in which most in situ tumors were likely to be screen-detected, the familial risks in women with both in situ and invasive were still comparable. This suggests that detection of in situ tumors, screen-detected or otherwise, could be informative for breast cancer prevention decisions for relatives of patients with breast carcinoma in situ. For example, women with family history of invasive breast cancer in first-degree relatives are advised to start mammography screening at a younger age [11]. This study found that, after age 35 years, the 10-year cumulative risk of breast cancer was comparable in women with family history of invasive breast cancer or breast carcinoma in situ and thus similar strategies could be recommended for the prevention of breast cancer in both populations.

## Conclusion

This study, which is based on high-quality data, provides population-level evidence that the risk of breast cancer in women whose relatives are affected with breast carcinoma in situ is rather similar to that in women whose relatives have invasive breast cancer. Thus, family history of breast carcinoma in situ should not be overlooked. Cancer prevention guidelines and recommendations for women with a family history of invasive breast cancer could also be applicable to women with family history of breast carcinoma in situ.

## Supplementary information

**Additional file 1 : Table S1.** Relative risk of breast carcinoma in situ in women with family history of invasive breast cancer or breast carcinoma in situ*.*

## Data Availability

All sharable data is already present within the manuscript and the Additional file 1, and no additional data is available. The entire nationwide datasets used for this study cannot be publicly shared because of data protection rules in Sweden.
