# Supplementary material for: Risk of invasive breast cancer in relatives of patients with breast carcinoma in situ: a prospective cohort study
Source: BMC Med. 2020 Nov 5;18:295. doi: 10.1186/s12916-020-01772-x (PMC7643418; doi:10.1186/s12916-020-01772-x)
Supplement: Supplementary file 1 — Additional file 1 : Table S1. Relative risk of breast carcinoma in situ in women with family history of invasive breast cancer or breast carcinoma in situ. [file 12916_2020_1772_MOESM1_ESM.docx]

**Risk of invasive breast cancer in relatives of patients with breast carcinoma *in situ*: A prospective cohort study**

Trasias Mukama, Mahdi Fallah^=^, Hermann Brenner, Xing Xu, Kristina Sundquist, Jan Sundquist, Elham Kharazmi^=^

**Additional information**

**Additional file 1: eResults**

***Familial risk of breast carcinoma in situ***

We also assessed and compared the risk of breast carcinoma *in situ* in women with family history of invasive breast cancer and in women with breast carcinoma *in situ* in first-degree relatives. Women with a history of invasive breast cancer in one first-degree relative had 1.7-fold (SIR=1.7, 95% CI: 1.7–1.8) increased risk of having a breast carcinoma *in situ* diagnosis compared to women without family history of any breast tumor. The risk of breast carcinoma *in situ* was similar (1.7, 1.4–2.1) in women with a history of breast carcinoma *in situ* in one first-degree relative (Additional file 1: Table S1).”

**Additional file 1: Table S1. Relative risk of breast carcinoma *in situ* in women with family history of invasive breast cancer or breast carcinoma *in situ***

| **Family history of breast tumor** | **Age at breast carcinoma *in situ* diagnosis in index woman (years)** | | | | | | | | | | | |
| --- | --- | --- | --- | --- | --- | --- | --- | --- | --- | --- | --- | --- |
|  | **All ages** | | |  | **<50** | | |  | **≥50** | | | |
|  | **Obs** | **SIR** | **95% CI** |  | **Obs** | **SIR** | **95% CI** |  | **Obs** | **SIR** | **95% CI** |  |
| **No family history of *in situ* or invasive** | 16,211 | Reference | |  | 4,820 | Reference | |  | 11,391 | Reference | |  |
| **1 FDR invasive** | 2,019 | **1.7** | 1.7–1.8 |  | 497 | **2.1** | 1.9–2.3 |  | 1,522 | **1.7** | 1.6–1.7 |  |
| **≥2 FDRs invasive** | 130 | **2.9** | 2.4–3.5 |  | 22 | **6.4** | 4.0–9.7 |  | 108 | **2.6** | 2.2–3.2 |  |
| **1 FDR *in situ*** | 110 | **1.7** | 1.4–2.1 |  | 28 | **1.9** | 1.3–2.8 |  | 82 | **1.6** | 1.3–2.0 |  |

Obs = Observed number of cases with breast carcinoma *in situ*; SIR = Standardized incidence ratio; FDR = First-degree relative; CI = Confidence interval; Bold values = Statistically significant. All SIRs were adjusted for age, socio-economic status, period, and region.
